# Mesenchymal stromal/stem cell therapy for radiation-induced salivary gland hypofunction in animal models: a protocol for a systematic review and meta-analysis

**DOI:** 10.1186/s13643-022-01943-2

**Published:** 2022-04-18

**Authors:** Per Marcus Jansson, Charlotte Duch Lynggaard, Amanda Fenger Carlander, Siri Beier Jensen, Bjarke Follin, Cecilie Hoeeg, Birgitte Saima Kousholt, Rasmus Tolstrup Larsen, Christian Grønhøj, Kathrine Kronberg Jakobsen, Susie Rimborg, Anne Fischer-Nielsen, Julia M. L. Menon, Christian von Buchwald

**Affiliations:** 1 Department of Otolaryngology and Audiology, Head and Neck Surgery, Rigshospitalet, Copenhagen University Hospital, Copenhagen, Denmark; 2 The Royal Library, Library of Copenhagen, University of Copenhagen, Copenhagen, Denmark; 3 Department of Clinical Medicine, University of Copenhagen, Copenhagen, Denmark; 4 Deparment of Dentistry and Oral Health, Aarhus University, Aarhus, Denmark; 5 Cardiology Stem Cell Centre, Rigshospitalet, Copenhagen University Hospital, Copenhagen, Denmark; 6 Aarhus University Group for Understanding Systematic Reviews and Meta-analyses in Translational Preclinical Science, Department of Clinical Medicine, Aarhus University, Aarhus, Denmark; 7 Department of Occupational Therapy and Physiotherapy, Rigshospitalet, Copenhagen University Hospital, Copenhagen, Denmark; 8 Section of Social Medicine, Department of Public Health, University of Copenhagen, Copenhagen, Denmark; 9 Department of Immunology, Rigshospitalet, Copenhagen University Hospital, Copenhagen, Denmark; 10 Systematic Review Center for Laboratory animal Experimentation (SYRCLE), Department for Health Evidence, Radboud University Medical Center, Nimegen, the Netherlands

**Keywords:** Systematic review, Radiotherapy, Xerostomia, Salivary gland hypofunction, Stem cells

## Abstract

**Background:**

Salivary gland (SG) hypofunction (objectively reduced saliva flow rate) and xerostomia (subjective sensation of dry mouth) are common and burdensome side effects of radiotherapy to the head and neck region. Currently, only sparse symptomatic treatment is available to ease the discomfort of xerostomia. The objective of this study is to assess the effect of mesenchymal stem cell (MSC) therapy on SG function after radiation-induced injury.

**Methods:**

This systematic review will include animal intervention studies assessing efficacy and safety of MSCs in treating radiation-induced SG hypofunction. The primary outcome is the effect of MSC administration on salivary flow rates (SFR), by comparing treated groups to control groups when available. Secondary outcomes are morphological and immunohistochemical effects as well as safety of MSC treatment. Electronic searches in MEDLINE (PubMed) and Embase databases will be constructed and validated according to the peer review of electronic search strategies (PRESS) and assessed by two independent researchers. Data from eligible studies will be extracted, pooled, and analyzed using random-effects models. Risk of bias will be evaluated with the Systematic Review Centre for Laboratory animal Experimentation (SYRCLE) risk of bias tool.

**Discussion:**

Thus far, critical appraisal of MSC therapy as an effective treatment for SG hypofunction caused solely by radiation injury has not been conducted. A summary of the existing literature on preclinical studies concerning this issue can provide valuable information about effectiveness, mode of action, and safety, allowing further optimization of preclinical and clinical trials.

**Systematic review registration:**

PROSPERO CRD42021227336

**Supplementary Information:**

The online version contains supplementary material available at 10.1186/s13643-022-01943-2.

## Background

Impaired salivary gland (SG) function and xerostomia (subjective sensation of dry mouth) are very common long-term side effects of radiation to the head and neck region [1]. Radiation therapy plays a key role in the curative treatment of most head and neck cancers, either independently or in adjunction to other treatment modalities, such as surgery and chemotherapy [2]. Although the emergent intensity-modulated radiation therapy (IMRT) allows a more precise delivery to target tumor tissue while to some extent sparing surrounding tissues, the SGs are often included in the radiation portal, leading to degeneration with long-term loss of function [3, 4]. In addition to the subjective sensation of dry mouth, decreased salivation predisposes to speech and swallowing problems, infections and tooth loss, and consequently poor quality of life. Only sparse symptomatic treatment is available yet, and no current regimens are aimed at restoring SG function [1, 5–7].

Accumulating literature on animal studies suggests that mesenchymal stromal/stem cell (MSC) transplantation therapy can restore radiation-injured SGs and hence can be a potentially curative treatment for SG hypofunction [8, 9]. Moreover, MSC therapy was shown to enhance the unstimulated salivary flow rate (SFR) in human participants in a randomized control phase I/II trial [10].

MSCs are multipotent adult cells capable of differentiating into all mesodermal lineages (i.e., osteoblasts, adipocytes, chondrocytes) in vitro. They have been isolated from numerous connective tissues and have been shown to enhance regeneration through their angiogenic, anti-inflammatory, and immune-modulatory properties [11–13].

Recent systematic reviews have addressed the potential value of MSCs as an effective treatment for SG hypofunction and xerostomia [8, 9, 14]. However, critical appraisal of its use in SG hypofunction caused solely by radiation damage has yet to be conducted. It is therefore of great importance to review preclinical studies on this issue in order to optimize further clinical trials and to favor its prospective implication in the curative treatment of radiation-induced SG hypofunction [15].

### Research questions

1. What impact does MSC therapy have on restoration of SG function after radiation-induced injury and hypofunction in animal models?
2. Is MSC therapy safe in terms of adverse events?

### Objectives

The main objective of the present study is to assess the effect of MSC therapy on restoring SG function after radiation-induced injury. By reviewing the literature on animal studies, we will examine if MSC therapy promotes changes in SFR as well as on SG morphology and in saliva composition. Furthermore, safety of the intervention will be analyzed by observing adverse events.

## Methods

### Protocol and registration

The systematic review protocol was developed by a research team of clinical and preclinical research scientists, experts in knowledge synthesis and translation, one expert in literature search, and experts in data management. It was designed according to the Preferred Reporting Items for Systematic Reviews and Meta-Analyses Protocols (PRISMA-P) [16]. A summary of the protocol is listed at PROSPERO. The final protocol is reported using the PRISMA guidelines (Additional file 1).

### Types of studies

This review will include animal intervention studies evaluating the efficacy and safety of MSC therapy for radiation-induced SG hypofunction. All studies addressing the above will be included, and it will be noted whether the studies were controlled, uncontrolled, randomized, and/or blinded. Human studies will be excluded in the systematic review; however, they will be mentioned/described in the introduction and/or discussion of the final manuscript to bring some background on the clinical implementation of MSC therapy for SG hypofunction. As far as we know, a single clinical trial was conducted on this topic [10].

### Types of animal models

We will include preclinical in vivo models of experimentally induced radiation damage of the major SGs that represent pathophysiological features of human SG hypofunction and xerostomia. No restrictions regarding induction of radiation damage (e.g., frequency, duration, power) will be applied. Eligible animal models include healthy mammals of both sexes and all ages. It will be noted if the models include the use of immunosuppressive drugs. In vitro studies will be excluded.

### Types of interventions

The assessed intervention will be MSC administration to animals exhibiting radiation-induced SG hypofunction, including intra-glandular MSC implantation, intravenous and/or intramuscular injections, and others. Studies with repeated dosing interventions will be included. MSCs may be autologous, allogeneic, syngeneic, or xenogeneic and derived from any organ or tissue (e.g., bone marrow, adipose tissue). It will be noted if MSCs have been preconditioned in any way, or if they have been labelled either genetically or by surface labelling. MSC secretome, exosomes, and treatment with parts of MSCs are also included. The time period between induction of radiation damage and MSC administration in included studies will be reported but will not be a restriction with regard to inclusion and exclusion of relevant studies. However, MSC administration must be subsequent to induction of radiation damage.

### Types of control comparisons

MSC-treated populations will be compared to untreated, vehicle-treated, or sham controls with experimentally induced radiation damage of the major SGs. Baseline controls are accepted and will be included in the (narrative) analysis. It will be noted if studies reported clearly defined controls.

### Types of outcome measures

#### Primary outcomes

The primary outcome is change in SG function measured by SFR subsequent to MSC administration.

#### Secondary outcomes

Defined a priori secondary outcomes are changes in SG morphology (e.g., weight, atrophy), SG histology including immunohistochemical appearance (e.g., cytoprotection, apoptosis, vascularity), changes in the composition of saliva (e.g., proteins, RNAs, inorganic ions), and circulating immune cells, SG paracrine effects, mode of action, and safety in terms of objective adverse events (e.g., animal mortality, morbidity, impaired physical activity, signs of pain or distress such as grimacing, flinching, writhing ) of MSC therapy.

#### Timing of outcome assessment

Outcomes will be assessed post-intervention. All time points will be extracted at data extraction and compared in the narrative synthesis. Only the final point will be kept for meta-analysis. If possible, subgroup analysis will be performed to compare the exposure and treatment patterns (e.g., frequency, time to treatment).

#### Electronic search strategies for identification of studies

Electronic search strategies were developed by the research group in collaboration with a medical information specialist. PubMed® and Embase® will be searched using Medical Subject Headings (MeSH), Emtrees, and text words relating to mesenchymal stem cells, salivary gland hypofunction, salivary gland damage, salivary gland dysfunction, radiation-induced salivary gland damage, or xerostomia. Search strings for the two databases were created, as shown below. Syntax and subject headings were adapted according to the database being searched. Search strategies will be validated using the peer review of electronic search strategies (PRESS) template by another member of the review team [17]. No restriction on publication date, publication type, or language will be applied. Apart from searching these databases, we will manually seek for further relevant articles by verifying reference lists of included papers.

#### Search string for PubMed

(((((("Stem Cells"[Mesh] OR "Stromal Cells"[Mesh] OR "Stem Cell Transplantation"[Mesh] OR Stem Cell*[Text Word] OR stromal cell*[Text Word] OR mesenchymal [Text Word] OR ASC*[Text Word] OR ADSC*[Text Word] OR MSC*[Text Word] OR BMSC*[Text Word] OR "cell therapy"[Text Word])) OR (("secretome"[Text Word] OR "exosome"[Text Word]))))) AND (((saliva*[Text Word] OR "Salivation"[Mesh] OR "Xerostomia"[Mesh] OR xerostomia [Text Word] OR hyposalivation [Text Word] OR "dry mouth"[Text Word] OR salivary gland*[Text Word] OR "salivary hypofunction"[Text Word] OR "intraglandular"[Text Word])) OR ((submandibular*[Text Word] OR parotid [Text Word])))) AND ("Radiotherapy"[Mesh] OR radiotherap*[Text Word] OR radiation*[Text Word] OR radio-induced [Text Word] OR irradiation*[Text Word] OR postradiation*[Text Word] OR chemoradiotherap*[Text Word] OR "Radiation Injuries"[Mesh] OR "Radiation"[Mesh])

### Search string for Embase

1. (stem cell/ or adipose derived stem cell/ or adult stem cell/ or exp mesenchymal stem cell/ or mononuclear stem cell/ or multipotent stem cell/)

2.stroma cell/

3. stem cell transplantation/ or allogeneic stem cell transplantation/ or autologous stem cell transplantation/ or mesenchymal stem cell transplantation/

4. (Stem Cell* or stromal cell* or mesenchymal or ASC* or ADSC* or MSC* or BMSC* or "cell therapy").mp. [mp=title, abstract, heading word, drug trade name, original title, device manufacturer, drug manufacturer, device trade name, keyword, floating subheading word, candidate term word]

5. 1 or 2 or 3 or 4

6. saliva/

7.

hyposalivation/

8.

xerostomia.mp. or exp xerostomia/

9.

salivation.mp. or exp salivation disorder/ or salivation/

10.

dry mouth.mp.

11.

salivary gland.mp. or salivary gland/

12.

(xerostomia or hyposalivation or "dry mouth" or salivary gland* or "salivary hypofunction" or "intraglandular" or saliva).mp. [mp=title, abstract, heading word, drug trade name, original title, device manufacturer, drug manufacturer, device trade name, keyword, floating subheading word, candidate term word]

13.

salivation.mp. [mp=title, abstract, heading word, drug trade name, original title, device manufacturer, drug manufacturer, device trade name, keyword, floating subheading word, candidate term word]

14.

11 or 12 or 13

15.

exp radiation/ or exp intensity modulated radiation therapy/ or exp radiation injury repair/ or radiation.mp. or exp radiation exposure/ or exp ionizing radiation/ or exp radiation injury/

16.

(radiotherap* or radiation* or radio-induced or irradiation* or postradiation* or chemoradiotherap*).mp. [mp=title, abstract, heading word, drug trade name, original title, device manufacturer, drug manufacturer, device trade name, keyword, floating subheading word, candidate term word]

17.

15 or 16

18.

5 and 14 and 17

19.

limit 18 to updaterange="oemezd (20200429195810-20200429195810]"

20.

("exosome" or "secretome").mp. [mp=title, abstract, heading word, drug trade name, original title, device manufacturer, drug manufacturer, device trade name, keyword, floating subheading word, candidate term word]

21.

4 or 20

22.

(submandibular* or parotid).mp. [mp=title, abstract, heading word, drug trade name, original title, device manufacturer, drug manufacturer, device trade name, keyword, floating subheading word, candidate term word]

23.

6 or 7 or 8 or 9 or 10 or 11 or 12 or 13 or 22

24.

17 and 21 and 23

#### Searching other resources

Three reviewers (PMJ, AFC, CH) will manually seek for further relevant articles by verifying reference lists of included papers as well as reviews that are excluded but identified during screening.

#### Data collection and analysis

The online software Covidence© (Covidence systematic review software, Veritas Health Innovation, Melbourne, Australia. Available at www.covidence.org) will be used for importing citations from literature searches, screening of abstracts and titles, screening of full texts, and extracting data. Duplicates will be removed using EndNote™. All analyses will be conducted in the statistical software R©.

#### Selection of studies and data extraction

Three researchers (AFC, CH, PMJ) will independently screen titles and abstracts, after which full-text articles meeting the inclusion criteria will be identified and reviewed. Inclusion criteria are as follows: animal intervention studies including mammals of both sexes and all ages, exposure of SGs to ionizing radiation, and MSC therapy of all administration routes and outcomes mentioned in “Types of outcome measures.” In vitro studies, treatments other than MSCs, MSC secretome and exosomes, and SG damage due to causes other than ionizing radiotherapy and nonrelevant outcomes will be excluded. Disagreements will be solved by consensus, and if no agreement can be reached, another reviewer (BF or CDL) will be consulted. Reasons for exclusion of potentially eligible studies will be recorded and presented in accordance with the PRISMA-P guidelines developed for proper reporting of clinical systematic reviews.

Study level characteristics will be extracted independently by two researchers: study characteristics (e.g., design), study population (e.g., species, race, sex, gene modifications), type of SG injury induction (e.g., radiation injury, radiation dose, timing of injury induction with regard to intervention), type of anesthesia, intervention, and comparison (e.g., MSC dose and administration route, number of treatments), preclinical endpoints (e.g., SFR, adverse events), and timing of endpoint measurement and follow-up time and deaths. Data will be extracted from text, tables, and graphs. Graphs data will be extracted using the digital ruler WebPlotdigitizer©. Discrepancies will be resolved by consensus or by a third member of the review team. Data extraction forms will be prepared a priori, and a calibration exercise will pilot five studies to refine the forms and ensure inter-rater consistency using Cohen’s kappa coefficient [18]. Inter-rater reliability for ordered (nonbinary or categorical outcomes) will be evaluated using appropriate methods (Pearson correlation for continuous data and Spearman correlation for ranked data).”

Foreign articles in Swedish, Danish, Norwegian, German, and French will be extracted by knowledgeable co-authors and collaborators. If an article in another language than the one mentioned is included, we will extract data as best as possible using Google translate. In the event where this could not be done, the article would be excluded from data analysis in post hoc. Data to be extracted are either continuous (e.g., SFR (ml/min)) or dichotomous (e.g., adverse events). In case of missing data, authors will be contacted by e-mail twice; if we receive no answer after the second e-mail, the authors will be considered unreachable.

#### Assessment of risk of bias in included studies

The methodological quality will be assessed independently by two researchers using the SYRCLE (Systematic Review Center for Laboratory animal Experimentation) risk of bias tool [19]. This includes assessment of domains related to selection bias, performance bias, detection bias, attrition bias, and reporting bias. For each included study, each domain will be scored as low, high, or unclear risk of bias. To address the effect of risk of bias and small study size, we will perform sensitivity analysis where we will remove high risk of bias studies and/or small study to the meta-analysis. A meta-analysis will be performed if there are at least two included studies. However, we will then emphasize that the results most likely are due to a low number of studies if that is the case and hence need critical appraisal. Discrepancies will be resolved by consensus or by a third member of the review team.

#### Description of reporting

The latest Animal Research: Reporting of In Vivo Experiments (ARRIVE) guidelines will be used to evaluate the quality of reporting in preclinical studies. The ARRIVE guidelines were developed by the National Centre for the Replacement, Refinement, and Reduction of Animals in Research (NC3Rs) to improve the transparent and comprehensive reporting of research methods and results for in vivo animal experiments [20].

#### Data analysis

A descriptive summary will be conducted for all outcomes. To evaluate the efficacy of MSCs in treating radiation-induced SG hypofunction, a random-effects meta-analysis adjusted to Hedge’s g will be conducted on SFR. We consider meta-analysis as feasible with a minimum of two studies. If possible, subgroup analyses will be conducted on studies using complete MSCs and studies using MSC secretome or exosomes. Subgroup analysis will also be considered on other heterogeneity factors such as species, strain, sex, age, radiation characteristics (dose, duration, frequency), MSC administration route, frequency of treatment, and time between radiation and treatment. To perform this analysis, we require at least two studies in different categories. If there are multiple time points, only the last one will be included in the meta-analysis. If all studies included in the quantitative synthesis report similar outcome measures for SFR, the pooled effect estimate will be quantified with a weighted mean difference with 95% confidence intervals (95% CI). If not, a standardized mean difference with 95% CIs will be used. To evaluate safety of the treatment, a random-effects meta-analysis will be conducted on adverse events. The pooled estimate will be quantified with risk ratios with 95% CIs. Heterogeneity of the study results will be investigated using Cochrane *Q*-test and quantified with *I*^2^ values.

## Discussion

Regenerative stem cell therapy is considered a prospective curative treatment for SG hypofunction and xerostomia. A large number of irradiated head and neck cancer patients worldwide are suffering from the harsh and long-lasting side effect of SG hypofunction and xerostomia, hence the importance of reaching a curative treatment.

Previous systematic reviews on preclinical trials have shown promising effects of MSC therapy on SG hypofunction [8, 9, 14]. However, these reviews have included SG hypofunction as a consequence of various causes, and therefore, different pathophysiological patterns could be expected. The effect of MSC therapy on SG hypofunction caused solely by radiation injury has not been evaluated.

Early phases of clinical trials on MSC treatment on radiation-induced SG hypofunction have recently been accomplished [10]. Progression of clinical research on the issue warrants further examination of efficacy and safety in animal models, supposedly improving methodology and standardizing procedures in order to manage radiation-induced SG hypofunction and xerostomia in a clinical setting.

## Supplementary Information

**Additional file 1.** Preferred Reporting Items for Systematic review and Meta-Analysis Protocols (PRISMA-P) 2015 checklist: recommended items to address in a systematic review protocol.

**Additional file 2.** A. Search string for PubMed. B. Search string for EMBASE.

## Data Availability

Not applicable

## Abbreviations

MSC: Mesenchymal stem cell
SG: Salivary gland
SFR: Salivary flow rate
PRESS: Peer review of electronic search strategies
SYRCLE: SYstematic Review Center for Laboratory animal Experimentation
PRISMA-P: Preferred Reporting Items for Systematic Reviews and Meta-Analyses Protocol
ARRIVE: Animal Research: Reporting of In Vivo Experiments
CI: Confidence interval
