# Supplementary material for: Mesenchymal stromal/stem cell therapy for radiation-induced salivary gland hypofunction in animal models: a protocol for a systematic review and meta-analysis
Source: Syst Rev. 2022 Apr 18;11:72. doi: 10.1186/s13643-022-01943-2 (PMC9016929; doi:10.1186/s13643-022-01943-2)
Supplement: Supplementary file 2 — Additional file 2. A. Search string for PubMed. B. Search string for EMBASE. [file 13643_2022_1943_MOESM2_ESM.docx]

**Additional File 2**

**A. Search string for PubMed**

(((((("Stem Cells"[Mesh] OR "Stromal Cells"[Mesh] OR "Stem Cell Transplantation"[Mesh] OR Stem Cell*[Text Word] OR stromal cell*[Text Word] OR mesenchymal[Text Word] OR ASC*[Text Word] OR ADSC*[Text Word] OR MSC*[Text Word] OR BMSC*[Text Word] OR "cell therapy"[Text Word])) OR (("secretome"[Text Word] OR "exosome"[Text Word]))))) AND (((saliva*[Text Word] OR "Salivation"[Mesh] OR "Xerostomia"[Mesh] OR xerostomia[Text Word] OR hyposalivation[Text Word] OR "dry mouth"[Text Word] OR salivary gland*[Text Word] OR "salivary hypofunction"[Text Word] OR "intraglandular"[Text Word])) OR ((submandibular*[Text Word] OR parotid[Text Word])))) AND ("Radiotherapy"[Mesh] OR radiotherap*[Text Word] OR radiation*[Text Word] OR radio-induced[Text Word] OR irradiation*[Text Word] OR postradiation*[Text Word] OR chemoradiotherap*[Text Word] OR "Radiation Injuries"[Mesh] OR "Radiation"[Mesh])

**B. Search string for EMBASE**

1.

(stem cell/ or adipose derived stem cell/ or adult stem cell/ or exp mesenchymal stem cell/ or mononuclear stem cell/ or multipotent stem cell/)

2.stroma cell/

3. stem cell transplantation/ or allogeneic stem cell transplantation/ or autologous stem cell transplantation/ or mesenchymal stem cell transplantation/

4.

(Stem Cell* or stromal cell* or mesenchymal or ASC* or ADSC* or MSC* or BMSC* or "cell therapy").mp. [mp=title, abstract, heading word, drug trade name, original title, device manufacturer, drug manufacturer, device trade name, keyword, floating subheading word, candidate term word]

5.

1 or 2 or 3 or 4

6.

saliva/

7.

hyposalivation/

8.

xerostomia.mp. or exp xerostomia/

9.

salivation.mp. or exp salivation disorder/ or salivation/

10.

dry mouth.mp.

11.

salivary gland.mp. or salivary gland/

12.

(xerostomia or hyposalivation or "dry mouth" or salivary gland* or "salivary hypofunction" or "intraglandular" or saliva).mp. [mp=title, abstract, heading word, drug trade name, original title, device manufacturer, drug manufacturer, device trade name, keyword, floating subheading word, candidate term word]

13.

salivation.mp. [mp=title, abstract, heading word, drug trade name, original title, device manufacturer, drug manufacturer, device trade name, keyword, floating subheading word, candidate term word]

14.

11 or 12 or 13

15.

exp radiation/ or exp intensity modulated radiation therapy/ or exp radiation injury repair/ or radiation.mp. or exp radiation exposure/ or exp ionizing radiation/ or exp radiation injury/

16.

(radiotherap* or radiation* or radio-induced or irradiation* or postradiation* or chemoradiotherap*).mp. [mp=title, abstract, heading word, drug trade name, original title, device manufacturer, drug manufacturer, device trade name, keyword, floating subheading word, candidate term word]

17.

15 or 16

18.

5 and 14 and 17

19.

limit 18 to updaterange="oemezd(20200429195810-20200429195810]"

20.

("exosome" or "secretome").mp. [mp=title, abstract, heading word, drug trade name, original title, device manufacturer, drug manufacturer, device trade name, keyword, floating subheading word, candidate term word]

21.

4 or 20

22.

(submandibular* or parotid).mp. [mp=title, abstract, heading word, drug trade name, original title, device manufacturer, drug manufacturer, device trade name, keyword, floating subheading word, candidate term word]

23.

6 or 7 or 8 or 9 or 10 or 11 or 12 or 13 or 22

24.

17 and 21 and 23
